# Association of metallothionein 2A rs10636 with low mean corpuscular volume (MCV), low mean corpuscular haemoglobin (MCH) in healthy Taiwanese

**DOI:** 10.1038/s41598-022-27304-6

**Published:** 2023-01-23

**Authors:** Rong-Fu Chen, Po-Ming Chen, Chau-Shiung Pan, Chieh-Cheng Huang, En-Pei Isabel Chiang

**Affiliations:** 1 Division of Plastic Surgery, Department of Surgery, Kaohsiung Medical University Hospital, Kaohsiung, 807 Taiwan, Republic of China; 2 Department of Food Science and Biotechnology, National Chung Hsing University, Taichung, Taiwan, Republic of China; 3 Research Assistant Center, Show-Chwan Memorial Hospital, Changhua, 500 Taiwan, Republic of China; 4 Department of Neurology, Show-Chwan Memorial Hospital, Changhua, Taiwan, Republic of China; 5 Department of Life Science, National Chung Hsing University, Taichung, 40402 Taiwan, Republic of China; 6 Innovation and Development Center of Sustainable Agriculture (IDCSA), National Chung Hsing University, Taichung, 402 Taiwan, Republic of China

**Keywords:** Gene expression, Genetic association study, Genetic markers

## Abstract

Human metallothionein-2A (MT2A) protein participates in metal homeostasis, detoxification, oxidative stress reduction, and immune defense. It decreases heavy metal ions and reactive oxygen species (ROS) during injury of cells and tissues. The single nucleotide polymorphisms at the MT2A gene have been associated in various human diseases including cancer. The current study aimed to elucidate associations between MT2A genotypes with the clinical, biochemical, and molecular characteristics that potentially related to lowered MT2A ex-pression. One hundred and forty-one healthy Taiwanese subjects were enrolled from Changhua Show-Chwan Memorial Hospital. Clinical, biochemical and molecular characteristics including the frequent minor allele SNPs, rs28366003 and rs10636, within the MT2A gene were determined. The genotype distribution of MT2A rs10636 fits the Hardy–Weinberg equilibrium. The significant associations with gradually decline of mean corpuscular volume (MCV) and mean corpuscular hemoglobin (MCH) were identified with MT2A rs10636 and rs28366003 using analysis of variance (ANOVA) with Tukey’s analysis as a post hoc test. We further validated the correlations between the expressions of genes in erythropoiesis, cholesterol synthesis, platelet synthesis, insulin with MT2A using the web-based Gene Expression Profiling Interactive Analysis (GEPIA) databases. The results revealed that hypoxia-inducible factor 1α (HIF-1α), erythropoietin (EPO), lipoprotein lipase (LPL), and lecithin-cholesterol acyltransferase (LCAT) mRNA ex-pression are significantly correlated with MT2A mRNA expression. In conclusion, these results suggested that genetic variations of MT2A rs10636 and rs28366003 might be an important risk factor for erythropoiesis in the Taiwanese general population.

## Introduction

Non-communicable chronic diseases result in a great health burden worldwide. In females, the causes for disability with the greatest age-standardized prevalence were oral disorders, headache disorders, and haemoglobinopathies and haemolytic anaemias in both 1990 and 2017. In males, the causes for disability with the greatest age-standardized prevalence were oral disorders, headache disorders, and tuberculosis that significantly contributed to the disability-adjusted life years^1,2^. Industrialization in Taiwan since the 1980s has drastically changed the eco-economic systems^3^; and exposure to surrounding ungreenness had been found to be positively associated with mortality in Taiwan^4^.

Low molecular mass (6–7 kDa) metallothioneins (MTs) are a family of metal-binding, cysteine-rich molecules which affect the homeostasis of intracellular metals such as cadmium, lead, mercury, zinc, and copper^5,6^. It has been identified as a potential biomarker for heavy metal toxicity as well as a set of genetic markers for certain pathologies^7,8^. Human metallothionein-2A (MT2A) protein participates in metal homeostasis, detoxification, oxidative stress reduction, and immune defense. It decreases heavy metal ions and reactive oxygen species (ROS) during injury of cells and tissues. Single nucleotide polymorphisms (SNPs) of the MT2A gene have been associated in various human pathological conditions. The MT2A SNPs may result in various cellular activities of metallothionein; they may alter the signaling of MT-dependent pathways that could steer the enhancement of tumor development and growth toward increased DNA damage, enhanced genomic instability, deregulated cell proliferation, inhibited apoptosis, and induced oxidative stress^5,8,9^. The polymorphism rs10636 in the MT2A gene has been reported to be essential for maintaining homeostasis under oxidative stress conditions in smokers^10^. SNPs of MT2A gene has also been found to modify lead body burden in workers chronically exposed this metal^11^, modulate the concentrations of the metal in the body and, consequently, adverse health effects induced by Pb exposure^11^. MT2A rs28366003 polymorphic allele was proposed to affect the MT2A gene expression in prostate that may be associated with some metal accumulation^12^.

A case–control study in the Chinese Han population indicated that the minor allele G of MT2A rs28366003 was related to an increased breast cancer risk. A significantly increased breast cancer risk with rs10636 polymorphism among homozygote and recessive models was also found. Further subgroup analysis of breast cancer patients showed that individuals with Scarff, Bloom and Richardson tumor grade (SBR) 1–2 had a higher expression of the minor allele of these two MT2A loci than SBR 3^13^.

Chromatin architecture and transcription factor binding regulate expression of erythrocyte membrane protein genes were examined previously, and MTA-2 binding and was found frequently in erythroid cell-expressed genes. Co-occupancy with FOG-1, SCL, and MTA-2 was found at all regions of GATA-1 binding, with co-occupancy of SCL and MTA-2 also found at regions of NF-E2 binding^14^. MT2A expression participates in the erythropoiesis, the regulation of complex genetic loci in erythroid, and nonerythroid cells. Numerous candidate regions for mutations associated with membrane-linked hemolytic anemia have been identified^14^.

Utilizing multiple bioinformatic tools, previous studies of ours explored and identified numerous novel biomarkers and explored the potential roles that they may participate in during human disease development^15–18^. The present study investigated and comprehensively explored associations between two SNPs of MT2A (rs10636 and rs28366003) and their predictive values for blood biochemical indexes in healthy Taiwanese population.

## Materials and methods

### Ethics statement and subjects studied

This study was approved by the Institution Review Board (IRB) of Changhua Show-Chwan Memorial Hospital, Taiwan (Document No. 1021203). Study inhabitants were randomly selected from the Changhua area. Inclusion into the study was based on the willingness (by the completion signing of the consent form) of the inhabitants to participate in the study, and willingness to provide the samples required, general good health determined by medical history and physical examination. One hundred and forty-one healthy Taiwanese subjects were enrolled from Changhua Show-Chwan Memorial Hospital. Clinical, biochemical and molecular characteristics including the frequent minor allele SNPs, rs28366003 (n = 141) and rs10636 (n = 121), within the MT2A gene were determined (the raw data is publicly available in the ‘Supplementary Information’ section).

### Blood collection and measurement

A maximum of 5 mL of venous blood samples were collected in serum separator tubes with gel and fluoride-EDTA tubes for hematology, biochemistry and glucose analysis. A total of 20 biochemical tests, including red blood cell [RBC], hemoglobin [Hgb], hematocrit [Hct], white blood cell [WBC], mean corpuscular volume [MCV], mean corpuscular hemoglobin [MCH], mean corpuscular hemoglobin concentration [MCHC], platelet [PLT], lymphocytes [Lym], segment [Seg], monocytes [Mono], eosinophils [Eosin], glutamic oxaloacetic transaminase [GOT], glutamic pyruvic transaminase [GPT], creatinine, cholesterol [CHOL], triglycerides [TG], fasting blood glucose [Glu-AC], uric acid [UA], high-density lipoprotein cholesterol [HDL-C], were measured by Changhua Show-Chwan Memorial Hospital Laboratory.

### Genotyping assays

MT2Ars28366003 and MT2Ars10636 have been identified in the 3’ untranslated region (3’UTR). Genomic DNA was isolated from heparin-anticoagulated blood samples using a standard phenol–chloroform extraction followed by 70% alcohol precipitation. Genotyping for the MT2A variants (rs10636 and rs28366003) were carried out using Custom TaqMan SNP kit (Thermo Fisher Scientific Inc., Waltham, MA USA). The probes were labeled with the TaqMan fluorescent dyes VIC and FAM. MT2A rs28366003 (C_1402094_10, Thermo Fisher Scientific Inc., Waltham, MA USA) sequence: (TGCTCCTGCTGCCCTGTGGGCTGTG[C/T]CAAGTGTGCCCAGGGCTGCATCTGC) and MT2A rs10636 (C_60284591_10, Thermo Fisher Scientific Inc., Waltham, MA USA) sequence: (GGATTTTTTATGTACAACCCTGACC[C/G]TGACCGTTTGCTATATTCCTTTTTC. The PCR was conducted in total volume of 15 µL using the following amplification protocol: denaturation at 95 °C for 10 min, followed by 40 cycles of denaturation at 94 °C for 20 s, followed by annealing and extension at 60 °C for one minute. After the PCR, the genotype of each sample was determined by measuring the allele-specific fluorescence in the ABI Prism 7500 Sequence Detection System, using SDS 1.1 software for allele discrimination (both Applied Biosystems, MA, USA). To validate the genotyping by real-time PCR analysis, 100 PCR products were subject to restriction fragment length polymorphism (RFLP) analysis with restriction enzymes and showed 100% identical result between these two genotyping systems.

### Statistical analyses

All data are expressed as the mean ± standard error of the mean (SEM). Differences between groups were evaluated using one-way analysis of variance (ANOVA), ANOVA followed by post-hoc Tukey’s multiple comparison tests. All analyses were performed using SPSS 18.0 (SPSS Inc. Chicago, IL, USA).

### Web server analysis

The correlations of expression of hypoxia-inducible factor 1-alpha (HIF1α), erythropoietin (EPO), lipoprotein lipase (LPL), lecithin-cholesterol acyltransferase (LCAT), glycoprotein Ib platelet subunit alpha (GP1BA), glycoprotein Ib platelet subunit beta (GP1BB), insulin (INS), and MT2A mRNA were calculated using Pearson’s correlation. Data obtained on 1 June 2022) (http://gepia2.cancer-pku.cn/#index).

### Institutional review board statement

The study was conducted in accordance with the Declaration of Helsinki, and approved by the Institutional Ethics Committee in Changhua Show-Chwan Memorial Hospital, Taiwan (Document No.: 1021203). Written informed consent to participate in observational studies was obtained from each patient.

### Informed consent statement

Informed consent was obtained from all subjects involved in the study.

## Results

### MT2Ars28366003 and MT2Ars10636 genotypes distributions in Taiwan

**Figure 1:**
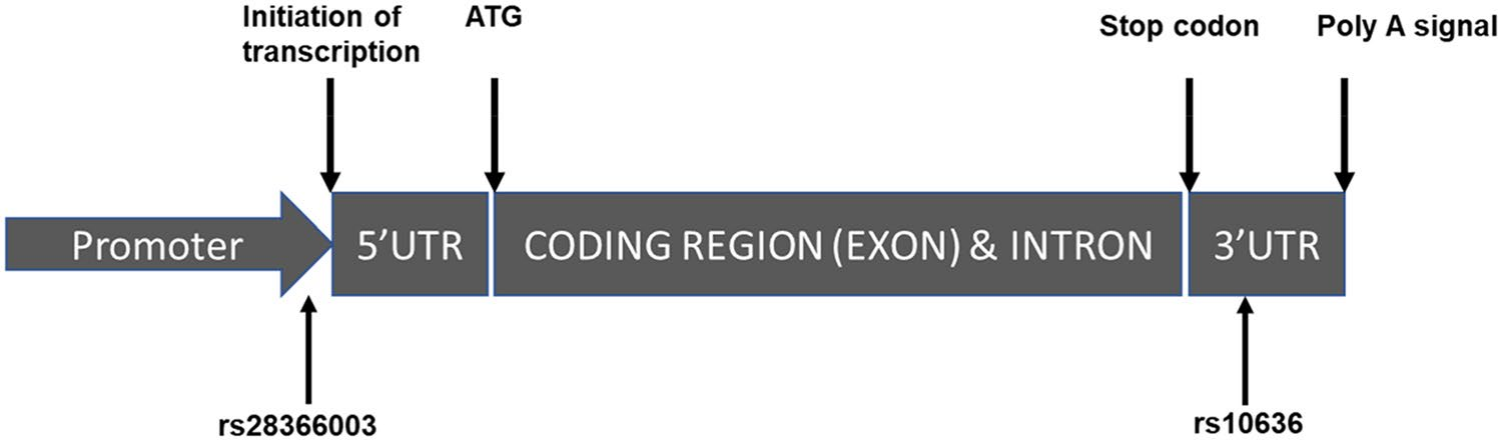
MT2A polymorphisms. Localization of clinically relevant single nucleotide polymorphisms (SNPs) in MT genes. SNP names according to NCBI dbSNP. UTR: untranslated region, ATG: start codon.

Two polymorphisms of MT2A (http://www.ncbi.nlm.nih.gov/snp/) have been reported with possible impact on physiological and pathophysiological processes^8^ (Fig. 1). Genotyping analysis of rs10636 (n = 121) and MT2Ars28366003 (n = 141) were examined with the Hardy–Weinberg equilibrium. The distributions of the clinical parameter and the MT2Ars28366003 and MT2Ars10636 polymorphisms are shown in Table 1. The rs10636 polymorphisms in the 121 subjects and the MT2Ars28366003 polymorphisms in 141 subjects were consistent with the frequencies expected from the Hardy–Weinberg Equilibrium with more than 5 subjects in every genotype group (chi-square test; p > 0.05, Table 1). The percentage of females and males in the subjects was 45% and 55%, respectively (Table 1). TaqMan SNP analysis was used to examine the MT2Ars28366003 and MT2Ars10636 polymorphisms. In 121 subjects, there were 90 subjects (74.4%) with wild-type alleles (A/A) of MT2Ars28366003, 30 subjects (25%) with heterozygote alleles (A/G) of MT2Ars28366003, and 1 subject (1%) with heterozygote variant alleles (G/G) of MT2Ars28366003 (Table 1). There were 63 subjects (52%) with wild-type alleles (G/G) of MT2Ars10636, 52 subjects (43%) with heterozygote alleles (G/C) of MT2Ars10636, and 6 subjects (5%) with heterozygote variant alleles (C/C) of MT2Ars10636 (Table 1).

**Table 1:** Demographics and MT2A genotypes of 141 residents and to determine whether observed genotype frequencies are consistent with Hardy–Weinberg equilibrium in Taiwan.

Demographics and clinical parameters	No. of patients	%	Variant allele frequency	*P*
**Age**				
Mean ± SD (y/o)	48 ± 18 (9–88)			
Gender				
Female	63	45		
Male	78	55		
**MT2Ars28366003**			0.13	0.75
AA	106	74		
AG	33	25		
GG	2	1		
**MT2Ars10636**			0.26	0.25
GG	63	52		
GC	52	43		
CC	6	5		
Not determination	20			

If *P* < 0.05, it is not consistent with Hardy–Weinberg equilibrium.

Not accurate if < 5 individual in any genotype group.

### MT2Ars10636 polymorphism is associated with low mean corpuscular volume (MCV), low mean corpuscular haemoglobin (MCH) of RBC and high platelet count

To evaluate the potential associations between MT2Ars10636 or MT2Ars28366003 with overall physical well-being, twenty biochemical indexes including RBC, Hgb, Hct, WBC, MCV, MCH, MCHC, PLT, Seg, Mono, Eosin, GOT, GPT, Creatinine, CHOL, TG, Glu-AC, UA, and HDL-C, were compared among different MT2A genotypes by ANOVA (Table 2).

**Table 2:** MT2Ars10636 and MT2Ars28366003 genotype distributions in demographics, clinical parameters of residents in Taiwan.

Demographics and clinical parameters	MT2Ars10636		Demographics and clinical parameters	MT2Ars28366003	
	Mean ± SD	P (One-way ANOVA)		Mean ± SD	*P*
RBC (10^6^/uL)		0.535	RBC (10^6^/uL)		0.217
GG	4.82 ± 0.56		AA	4.89 ± 0.63	
GC	4.91 ± 0.68		AG	4.74 ± 0.41	
CC	5.09 ± 0.87		GG	5.38 ± 0.16	
Hgb (g/dL)			Hgb (g/dL)		0.138
GG	14.53 ± 1.49	0.086	AA	14.46 ± 1.59	
GC	14.23 ± 1.62		AG	14.08 ± 1.36	
CC	13.10 ± 1.75		GG	12.65 ± 1.20	
HCT (%)		0.175	HCT (%)		0.147
GG	43.14 ± 3.98		AA	43.01 ± 4.13	
GC	42.53 ± 4.14		AG	42.24 ± 3.31	
CC	39.97 ± 4.41		GG	39.00 ± 2.96	
WBC (10^3^/uL)		0.405	WBC (10^3^/uL)		0.622
GG	5.98 ± 1.29		AA	6.10 ± 1.33	
GC	6.29 ± 1.64		AG	6.38 ± 1.91	
CC	6.60 ± 1.96		GG	6.50 ± 4.10	
MCV (fL)		**0.009**	MCV (fL)		**0.031**
GG	89.81 ± 5.72		AA	88.51 ± 7.45	
GC	87.29 ± 8.21		AG	89.24 ± 4.91	
CC	80.67 ± 15.23		GG	75.00 ± 28.28	
MCH (pg)		**0.002**	MCH (pg)		0.050
GG	30.21 ± 2.12		AA	29.72 ± 2.71	
GC	29.16 ± 2.94		AG	29.72 ± 2.07	
CC	26.35 ± 5.26		GG	24.95 ± 9.82	
MCHC (g/dL)		**0.045**	MCHC (g/dL)		0.323
GG	33.65 ± 0.93		AA	33.59 ± 1.01	
GC	33.41 ± 0.93		AG	33.30 ± 0.92	
CC	32.73 ± 0.89		GG	33.30 ± 0.56	
PLT (10^3^/uL)		**0.003**	PLT (10^3^/uL)		0.068
GG	220.41 ± 46.73		AA	226.25 ± 50.98	
GC	235.08 ± 53.36		AG	242.38 ± 51.47	
CC	293.33 ± 64.85		GG	295.50 ± 142.12	
Lym (%)			Lym (%)		0.993
GG	32.95 ± 7.51	0.760	AA	32.38 ± 6.76	
GC	32.08 ± 6.45		AG	32.22 ± 7.58	
CC	31.50 ± 8.60		GG	32.50 ± 16.26	
Seg (%)		0.501	Seg (%)		0.997
GG	57.68 ± 7.26		AA	58.70 ± 6.90	
GC	59.14 ± 7.11		AG	59.63 ± 7.93	
CC	59.83 ± 7.78		GG	59.00 ± 14.14	
Mono (%)		0.216	Mono (%)		0.349
GG	6.33 ± 1.64		AA	6.15 ± 1.52	
GC	5.86 ± 1.30		AG	5.97 ± 1.25	
CC	6.50 ± 1.76		GG	7.50 ± 2.12	
Eosin (%)		0.626	Eosin (%)		0.317
GG	2.98 ± 1.73		AA	2.70 ± 1.65	
GC	2.92 ± 3.06		AG	3.16 ± 3.50	
CC	2.00 ± 0.89		GG	1.00 ± 0.00	
GOT (U/L)		0.852	GOT (U/L)		0.615
GG	22.03 ± 7.27		AA	22.47 ± 8.95	
GC	22.38 ± 9.72		AG	23.74 ± 10.27	
CC	20.33 ± 8.33		GG	18.00 ± 5.65	
GPT (U/L)		0.384	GPT (U/L)		0.277
CC	24.42 ± 14.44		AA	25.99 ± 15.83	
CG	27.18 ± 18.47		AG	31.16 ± 28.08	
GG	18.50 ± 9.90		GG	14.50 ± 6.36	
Creatinine (mg/dL)		0.246	Creatinine (mg/dL)		0.413
GG	1.03 ± 0.20		AA	1.03 ± 0.24	
GC	1.02 ± 0.30		AG	0.96 ± 0.20	
CC	0.85 ± 0.19		GG	1.05 ± 0.21	
CHOL (mg/dL)		0.057	CHOL (mg/dL)		**0.004**
GG	179.40 ± 31.27		AA	179.90 ± 29.28	
GC	192.82 ± 35.76		AG	201.13 ± 39.22	
CC	168.83 ± 29.38		GG	164.50 ± 23.33	
TG (mg/dL)		0.647	TG (mg/dL)		0.383
GG	122.68 ± 100.74		AA	122.38 ± 86.05	
GC	134.59 ± 88.29		AG	145.00 ± 109.86	
CC	101.67 ± 69.78		GG	81.00 ± 9.89	
Glu-AC (mg/dL)		0.023	Glu-AC (mg/dL)		0.558
GG	103.37 ± 17.24		AA	101.35 ± 16.58	
GC	96.02 ± 9.04		AG	98.03 ± 10.63	
CC	96.17 ± 12.70		GG	104.50 ± 30.40	
UA (mg/dL)		0.825	UA (mg/dL)		0.429
GG	6.12 ± 1.45		AA	6.14 ± 1.43	
GC	6.08 ± 1.50		AG	6.09 ± 1.50	
CC	5.73 ± 1.47		GG	4.80 ± 0.70	
HDL-C (mg/dL)		0.387	HDL-C (mg/dL)		0.294
GG	56.23 ± 12.14		AA	56.22 ± 12.12	
GC	56.04 ± 12.82		AG	59.13 ± 13.49	
CC	63.33 ± 11.78		GG	66.50 ± 6.36	

*RBC* Red blood cell, *HgB* Hemoglobin, *Hct* Hematocrit, *WBC* White blood cell, *MCV* Mean corpuscular volume, *MCH* Mean corpuscular hemoglobin, *MCHC* Mean corpuscular hemoglobin concentration, *PLT* Platelet, *Lym* Lymphocytes, *Seg* Segment, *Mono* Monocytes, *Eosin* Eosinophils, *GOT* Glutamic oxaloacetic transaminase, *GPT* Glutamic pyruvic transaminase, *CHOL* Cholesterol, *TG* Triglycerides, *Glu-AC* Fasting blood glucose, *UA* Uric acid, *HDL* High-density lipoprotein cholesterol.

Significant values are in [bold].

**Figure 2:**
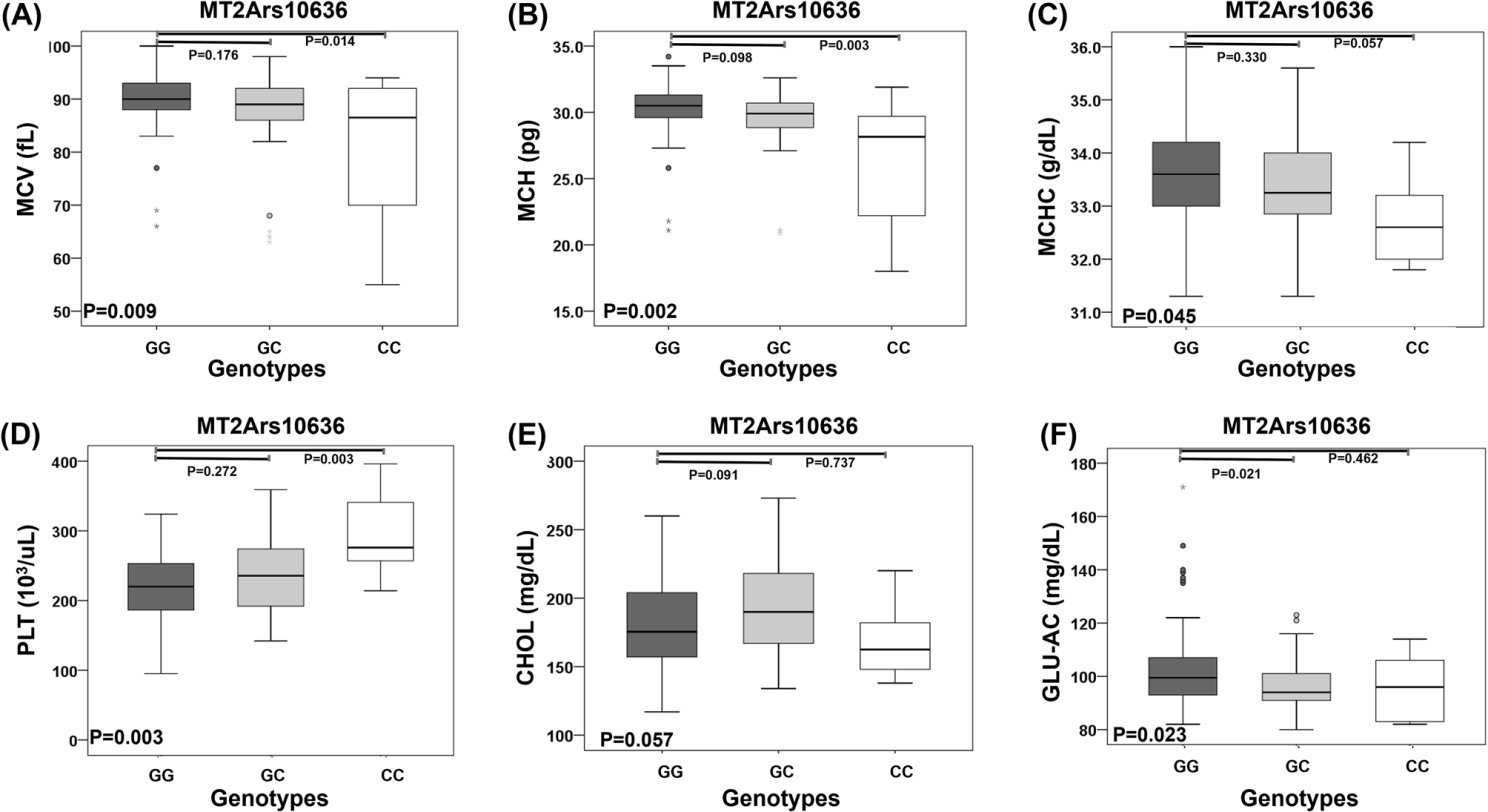
Associations of single point SNPs of MT2A with MCV, MCH, MCHC, PLT, CHOL, and GLU-AC. Data are expressed as mean ± SD. *P* value shows the comparison among three single point SNPs by ANOVA in the genotypes.

The subjects were generally in good health which was determined by medical history and physical examination. With respect to possible presence of anemia, no subjects had MCHC below 31%. Among the 141 subjects, 8.3% (n = 10) had MCV below 80 fL; 8.3% had MCH below 26 pg (n = 10); and no subjects had MCHC below 31%. These data indicated that most subjects were not anemic. Table 3 showed the characteristics and the distributions of blood biochemical indices among different genotypes. The result showed that MT2Ars10636 genotypes were significantly associated with MCV, MCH, MCHC, PLT, CHOL, and Glu-AC levels. ANOVA indicated that MT2Ars10636 is a significant determinant for the mean of MCV (*P* = 0.009, Fig. 2A), MCH (*P* = 0.002, Fig. 2B), MCHC (*P* = 0.045, Fig. 2C), PLT (*P* = 0.003, Fig. 2D), cholesterol (*P* = 0.0057, Fig. 2E), and fasting glucose levels (Glu-AC, *P* = 0.023, Fig. 2F). Post-hoc tests indicated further showed that mean MCV in subjects with MT2Ars10636 CC genotype was significantly lower than that in subjects with MT2Ars10636 GG (*p* = 0.014, Fig. 2A). The mean MCH level in subjects with MT2Ars10636 CC genotype was significantly lower than that in subjects with the MT2Ars10636 GG (*P* = 0.003, Fig. 2B). The mean MCHC level in subjects with MT2Ars10636 CC genotype was lower than that in subjects with the MT2Ars10636 G allele (*P* = 0.057, Fig. 2C). On the other hand, the mean PLT counts in in subjects with MT2Ars10636 CC genotype was significantly higher than that in subjects with the MT2Ars10636 G allele (*P* = 0.003, Fig. 2D). The mean fasting blood glucose (GLU-AC) in subjects with MT2Ars10636 GC genotype was significantly lower than that in subjects with MT2Ars10636 GG (*P* = 0.021, Fig. 2F).

**Table 3:** Characteristics of blood biochemical indices in the examined residents in Taiwan.

Measurements (normal range)	MT2Ars10636 genotype			
	GG (n = 63) n (%)	GC (n = 5 2) n (%)	CC (n = 6) n (%)	*P* (Chi-Square)
**MCV (80–100) (fL)**				**0.047**
> 100	0 (0)	0 (0)	0 (0)	
80–100	60 (95)	47 (90)	4 (66)	
< 80	3 (5)	5 (10)	2 (34)	
**MCH (26–34) (pg)**				**0.020**
> 34	0 (0)	0 (0)	0 (0)	
26–34	61 (94)	47 (90)	3 (50)	
< 26	2 (5)	5 (10)	3 (50)	
**MCHC (31–37) (g/dL)**				NA
> 37	0 (0)	0 (0)	0 (0)	
31–37	63 (100)	52 (100)	6 (100)	
< 31	0 (0)	0 (0)	0 (0)	
**PLT (150–400) (10**^**3**^**/uL)**				
> 400	0 (0)	0 (0)	0 (0)	0.702
150–400	59 (93)	50 (96)	6 (100)	
< 150	4 (7)	2 (4)	0 (0)	

Significant values are in [bold].

### MT2A mRNA expression is positively with HIF1α and EPO mRNA expression in whole blood tissue

The MT2A gene expression is regulated by miR-23a that can bind to the 3’UTR of this gene^19^. MT2A has been predicted to be targeted by 14 miRNAs including miR-23, miR-376, miR-12130, miR-493, miR-382, miR-3190, miR-3613, miR-6823, miR-3192, miR-4668, and miR-4795 that could distinctly decrease MT2A expression by RNA degradation or RNA translation (http://mirdb.org). (http://asia.ensembl.org/Homo_sapiens/Gene/Sequence? db = core; g = ENSG00000278799; r = 3:48,549,961–48,550,021; t = ENST00000622118). Based on the above bioinformation, the MT2Ars10636 SNP is potentially critical for binding of these miRNAs for MT2A RNA degradation or RNA translation. The results from this study confirmed that MT2A is a potential target of miR-23a and that miR-23a might play a role in regulating MT2A expression.

**Figure 3:**
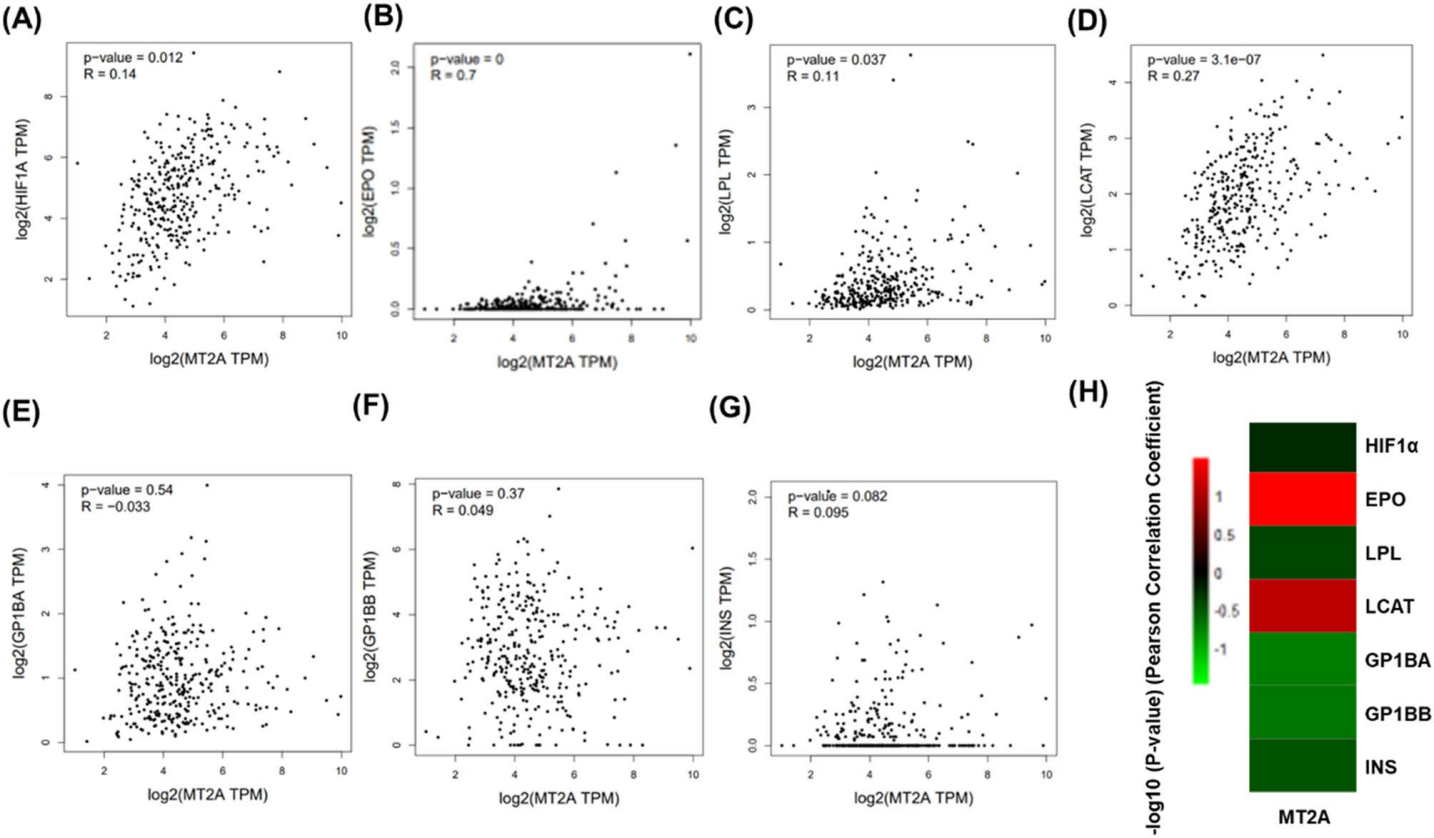
The direct correlation between RNA (TPM) in the whole blood tissue. (**A**–**F**) Pearson’s correlation elucidated the associations between HIF1α, EPO, LPL, LCAT, GP1BA, GP1BB INS, and MT2A mRNA expression, respectively. (**G**) Heatmap of Pearson’s correlations, shown by calculating (− log10 (*p* value)) for HIF1α, EPO, LPL, LCAT, GP1BA, and GP1BB use with MT2A. (**H**) Heatmap of Pearson’s correlations, shown by calculating (−Log10(*p*-value)) for MT2A use with HIF1α, EPO, LPL, LCAT, GP1BA, GP1BB, and INS.

The current study further investigated whether MT2A expression is associated with erythropoiesis genes (HIF1α, and erythropoietin [EPO]), cholesterol synthesis genes (LPL and LCAT), platelet synthesis genes (GP1BA and GP1BB), and INS (insulin) mRNA expression in whole blood tissues by web server analysis (http://gepia2.cancer-pku.cn/#index) (Fig. 3). The results revealed that HIF1α, EPO, LPL, and LCAT were significantly associated with MT2A expression (Fig. 3A–G), and the correlation of MT2A and EPO was conspicuous of red pixel in the heatmap (Fig. 3H).

## Discussion

The present study discovered the associations between MT2Ars10636 with MCV, and MCH, MCHC, in healthy Taiwanese population. Compared to the subjects with the MT2Ars10636 GG genotype, subjects with MT2Ars10636 CC genotype had significantly smaller red cells, less hemoglobin per red cell, and less hemoglobin per unit volume of the red cells. These results suggest that genetic variation of MT2Ars10636 might be a determining factor for erythropoiesis.

MCV can determine the classification of anemia and is useful for calculating the red blood cell distribution width (RDW)^20^. The red cell volume distribution width-standard deviation (RDW-SD) parameter is the best hematology index to predict the severity of COVID-19 patients and can be used as a useful indicator to help prevent and control the epidemic^21^ and a variety of hematologic changes are associated with the severity and clinical outcome of recovered COVID-19 patients^22^.

Inverse associations between certain hematological parameters and all-cause mortality among men have been reported^23^. The association between hematological parameters (MCV, MCH, and MCHC) and all-cause mortality was explained by a difference in iron metabolism, iron status, hormone regulations, or the occurrence of some diseases such as anemia of chronic disease, sideroblastic anemia, and thalassemia^23^. In adults, red blood cells are produced mainly in the bone marrow, and hormone erythropoietin (EPO) produced in the kidneys helps promote the production of red blood cells^24^. Using bioinformatic approach, we found that MT2A mRNA expression was highly correlated with EPO mRNA expression (Fig. 3B, r = 0.7, *p* < 0.00001, and 3H).

A study revealed that PLT to MCH ratio (PLT/MCH) can be used to distinguish between combined iron and vitamin B12 deficiency and uncomplicated iron deficiency^25^, suggesting that this parameter has good value for iron deficiency anemia diagnosis. As MT2Ars10636 CC subjects had significantly lower MCH and higher platelet counts (Fig. 2C–D), genetic variations of MT2A also deserve to be taken into consideration in iron deficiency anemia. The mechanism behind the altered platelet counts and MT2A function is unknown. Despite that ANOVA and post-hoc Tukey’s multiple comparison discovered a significantly higher mean platelet counts in MT2Ars10636 CC subjects compared to that of the GG subjects, MT2A mRNA expression was not associated with the expression levels of platelet activation-related gene GP1BA (Fig. 3E) or GP1BB (Fig. 3F). Nevertheless, genetic variations of MT2A deserve to be taken into consideration in the evaluation of thrombotic risk. Gene mapping showed that a large conserved linkage group exists on human chromosome 16q, including the loci for lecithin: cholesterol acyltransferase (LCAT), and metallothionein1 and 2 (Mt-1, -2)^26^. This may help partially explain the relationship between MT2A and LCAT mRNA levels observed in GEPIA.

MT has the function to protect vascular cells from apoptosis under hypoxia. Higher expression of HIF-1α and MT were reported in varicocele and varicose veins; hence MT proteins have been proposed to decrease vascular cell apoptosis and contribute to the dilated and thickened walls of varicocele and varicose veins^27^. On the other hand, over-expressions of HIF-1α, metallothionein and SLUG have been associated with high tumor-node-metastasis stage and lymph node metastasis in papillary thyroid carcinoma^28^; hence the evaluation of HIF-1α and MT may be useful in predicting the risk of lymph node metastasis and high tumor-node-metastasis stage and be associated with human cancer microenvironment.

In our study, GLU-AC was found significantly higher in subjects with MT2Ars10636 homozygous G allele (96.17 ± 12.7 mg/dL, mean ± SEM) (Table 2). This finding may imply a link between genetic variations of MT2A and the risk of diabetes. Metallothionein 2a gene expression was reported to be increased in subcutaneous adipose tissue of type 2 diabetic patients^29^. Exploring GEPIA database we discovered a mild correlation between mRNA expression of MT2A and insulin (Fig. 3, *p* = 0.082). These findings suggest that up-regulation of metallothionein gene might be involved in insulin resistance through the modulation of insulin action in type 2 diabetes. Consistently, a previous study showed a significant positive correlation between glycohemoglobin (HbA1c) value with Hb, HCT, and MCHC in non-diabetic pregnant women^30^.

Metallothionein was found to rescue HIF-1α transcriptional activity in cardiomyocytes under diabetic conditions^31^, and activation of HIF-1α by metallothionein was proposed to contribute to cardiac protection in the diabetic heart^32^. Moreover, high glucose was found to increase metallothionein expression in renal proximal tubular epithelial cells^33^. In rats with streptozotocin-induced diabetes, MT-1/-2 expression was gradually and dramatically increased in the proximal tubular epithelial cells^33^. It was proposed that the MT-1/-2 induction by high glucose exposure in renal proximal tubular epithelial cells may act as an antioxidant to protect the kidney from oxidative stress. Therefore, results from this study suggest that the C allele of MT2Ars10636 may be a favorable factor for diabetic patients, and that MT2A may offer a novel therapeutic target against diabetic nephropathy. In smokers with Crohn's disease, MT2A was found to be significantly up-regulated in the specimens with inflammatory activity of the descending colon^34^. The present study did not have smoking information on the participants; nor did it have access to the Taiwanese healthcare system due to the confidentiality. Hence, the present study has some limitations with respect to the potential factors that may affect the outcomes including smoking history, dietary patterns, physical activities, or comorbidities. Nevertheless, the study still clearly demonstrated that genetic variations of MT2A rs10636 is an important risk factor for erythropoiesis in the Taiwanese general population.

In conclusion, our results revealed that genetic variations of MT2A are important marker for erythropoiesis, and it that may provide clues for the common mechanisms related to initial insults to erythropoiesis in various human diseases (Supplementary information).

## Supplementary Information

Supplementary Information.

## Data Availability

The data presented in this study are available on request from the corresponding author.
